# Oncogenic proteome of pancreatic cancer extracellular vesicles: sodium/myo-inositol cotransporter as a potential marker

**DOI:** 10.1038/s41392-025-02232-9

**Published:** 2025-05-07

**Authors:** Arunima Panda, Krish Ragunath, Marina Pajic, David W. Greening, Marco Falasca

**Affiliations:** 1 Curtin Medical Research Institute, Curtin University, Perth, WA 6102 Australia; 2 Department of Gastroenterology and Hepatology, Royal Perth Hospital, Perth, WA 6100 Australia; 3 School of Clinical Medicine, Faculty of Medicine, University of New South Wales (UNSW) Sydney, Sydney, NSW Australia; 4 Translational Oncology Program, Garvan Institute of Medical Research, NSW Sydney, 2010 Australia; 5 Baker Heart and Diabetes Institute, Melbourne, VIC 3004 Australia; 6 Baker Department of Cardiometabolic Health, University of Melbourne, Melbourne, VIC Australia; 7 Baker Department of Cardiovascular Research Translation and Implementation, La Trobe University, Melbourne, VIC Australia; 8 Department of Medicine and Surgery, University of Parma, Parma, 43125 Italy; 9 Present Address: Curtin Medical Research Institute, Curtin University, Perth, WA 6102 Australia

**Keywords:** Gastrointestinal cancer, Prognostic markers

**Dear Editor**,

Small extracellular vesicles (sEVs) are membranous nanovesicles involved in intercellular communication that carry distinct cell-derived molecular cargo.^1^ We previously characterised sEVs from human non-malignant pancreatic duct cells (HPDE, hTERT–HPNE) and from PDAC cells (AsPC-1, BxPC-3 and MIA PaCa-2)^2^ and identified protein cargo-specific to cancer-associated sEVs.^2^ Among the proteins uniquely expressed in cancer sEVs but not in those from non-malignant cells, we focused on SLC5A3, also known as SMIT1 (sodium-coupled Myo-inositol transporter-1). SLC5A3 plays a crucial role in the transport of myo-inositol and is functionally implicated in cancer development and tumorigenesis.^3,4^ Notably, we identified sEV-associated SLC5A3 as an unfavourable prognostic marker in pancreatic ductal adenocarcinoma (PDAC); however, its precise role in cancer remains unexplored. We employed multi-omics (of cancer cells, derived biofluids, and isolated sEVs) to identify the biomarker potential of SLC5A3 in pancreatic cancer detection. To study the role of sEVs in pancreatic cancer, we selected a panel of 8 PDAC patient-derived cell lines (PDCL) containing the four PDAC subtypes based on variations in the chromosomal structure.^5^ We selected four genetic variant subtypes: stable (TKCC-06, −15), focal or locally rearranged (TKCC-07, −26), scattered (TKCC-05, −19) and unstable (TKCC 02, −27), in addition to cancer subtypes with differences in metastatic capacity and gemcitabine resistance (Fig. 1a). First, we isolated sEVs from eight PDAC PDCLs and MIA PaCa-2 cell models using differential ultracentrifugation. TKCC-26 and TKCC-27 cells were excluded due to insufficient protein yield in sEV samples for analysis. EV characterisation was performed based on morphology (transmission electron microscopy), particle size/diameter range (single nanoparticle tracking analysis), and sEV marker proteins (western immunoblotting and proteomic profiling) (Supplementary Information).

**Fig. 1:**
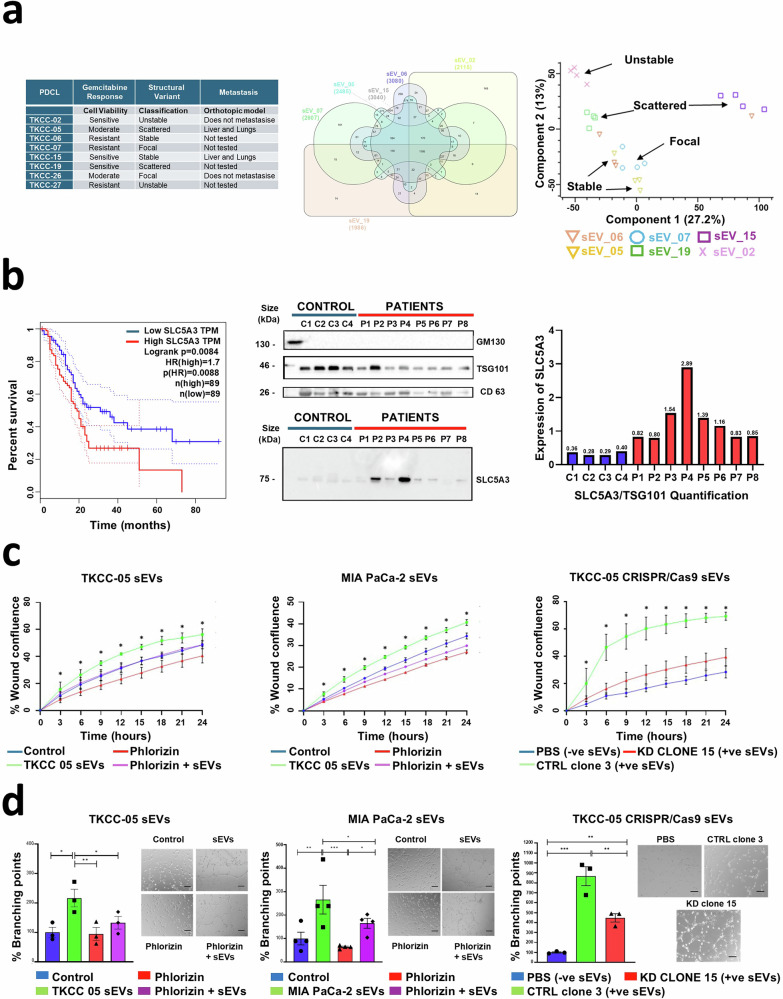
Comprehensive proteomic profiling of sEVs. **a** Key characteristics of patient-derived cell lines used in this study. A six-way Venn distribution of proteins identified in PDAC/TKCC derived sEVs, with 1195 proteins associated in all six sEVs models (TKCC 02, 05, 07, 06, 15 and 19), while each model has its distinct set of sEV proteins (TKCC 05- 273, TKCC 07- 161, TKCC 06- 258, TKCC 15- 434, TKCC 02- 149 and TKCC 19- 74 proteins). Correlation expression heatmap of PDAC-derived sEVs for each biological sample. **b** Effect of SLC5A3 expression on pancreatic cancer survival. Survival probability over time in months is presented for patients with high (*n* = 89) and low (*n* = 89) SLC5A3 expression. Data were sourced from the gene expression profiling interactive analysis (GEPIA). Western blot analysis of sEVs isolated from the blood of pancreatic cancer patients and healthy controls showing the levels of SLC5A3. Graph showing the Western blot quantification of SLC5A3 versus TSG101 in controls and patients. The quantification has been performed blindly by an independent scientist and refers to the blot shown in the figure. **c** PDAC- and PDCL- (TKCC) derived sEVs treatment promotes migration in human immortalised pancreatic cancer-associated fibroblasts (CAFs). CAF cells were treated with 0.05 µg/µl of TKCC-05 and MIA PaCa-2 sEVs. Treatment with TKCC-05 and MIA PaCa-2 sEVs significantly increases migration in CAFs. Pharmacological inhibition of SLC5A3 using 10 µM Phlorizin downregulates the function indicating the role of SLC5A3 in migration. CAFs cells treated with SLC5A3 KO sEVs showed significantly less migration compared to those treated with SLC5A WT sEVs. Data presented in mean ± SEM. *p value <0.05. Performed using scratch wound assay and monitored and evaluated with Operetta CLS. **d** PDAC- and PDCL- (TKCC) derived sEVs treatment promote tubulogenesis in HMEC-1 cells. Pharmacological inhibition of SLC5A3 using 10 µM Phlorizin downregulates sEVs-induced tubulogenesis. HMEC-1 cells treated with SLC5A3 KO sEVs showed significantly less tubulogenesis compared to those treated with SLC5A WT sEVs. Data were presented as the mean of three independent experiments run in triplicate ± SEM. Statistical significance was determined by one-way ANOVA. *p value <0.05. **c** Performed using in vitro branching tubulogenesis and monitored and evaluated with Incucyte® Live-Cell Analysis System (scale bar 50 μm)

Mass spectrometry (MS)-based proteomic analysis revealed 3715 cellular and 4496 sEV proteins across all sample groups. We next questioned whether the composition of sEVs differed between PDAC PDCLs (i.e. oncogenic signature). For sEVs, proteomic profiling identified a total of 4496 proteins, and a range of 1986–3080 proteins across specific cell models) (Fig. 1a). Proteins distributed across sEVs from different cancer cell models are highlighted (Fig. 1a), with 1195 proteins commonly identified (TKCC 02, 05, 06, 07, 15, 19), representing a conserved signature associated with PDAC-derived sEVs. Additionally, each model has a distinct set of proteins (e.g., TKCC 05 273 unique proteins, TKCC 07 161 unique proteins), reflecting the heterogeneity of sEV proteomes based on the source of different PDAC models. Further, principal component analysis of PDAC-derived sEVs (with respective biological replicates) highlights distinct clustering patterns associated with cell models based on their genomic stability/instability and metastatic potential (Fig. 1a). These differences in proteome highlight the potential of sEV proteome profiling in distinguishing stable and unstable genomic subtypes, as well as those PDCLs with varying metastatic capabilities. We further highlight that sEVs from all cancer models reflect sEV cargo distinct across all cell lines. Interestingly, sEV samples from cells with specific categories regarding genomic instability cluster independently. TKCC 02, PDCL characterised by an unstable genome, is distinct from the others regarding their sEV proteome content. Further, models characterised by the scattered genome (TKCC-19, TKCC-15) are also grouped but separated from the focal (TKCC-07) and stable (TKCC-05 and TKCC-06) subsets. These findings suggest that the degree of genomic instability of the source cells impacts the proteomic cargo of these sEVs. We identify protein networks associated with inositol metabolism and transport (Supplementary Information), including the inositol transporter SLC5A3 that correlates with poor prognosis. Integrative protein-protein (STRING) and regulatory network analyses (KEA3) revealed a core subset of co-transporters and metabolic regulatory proteins that interact with SLC5A3, and signalling pathways and downstream regulatory targets influenced by SLC5A3, including receptor kinases ERBB2 and GRK2. Both proteins are associated with cancer modulation and show elevated expression in pancreatic cancer, as well as in pan-cancer markers, based on protein and RNA expression data (Supplementary Information).

Further, to assess the impact of SLC5A3 on PDAC patient survival, we analysed datasets from Gene Expression Profiling Interactive Analysis (Fig. 1b). We highlight that high SLC5A3 expression is strongly associated with poor clinical outcome, confirming its status as a potential unfavourable prognostic marker in PDAC.

We performed proteome analysis of plasma-derived sEVs (eight PDAC patients and four healthy controls (Fig. 1b). We demonstrate that SLC5A3 expression is elevated in sEV-derived PDAC patients relative to non-disease (highly elevated for patients 2 and 4). To attenuate SLC5A3 function, we employed two approaches: pharmacological inhibition using the SLC5 family inhibitor phlorizin (10 μM; Fig. 1c) and CRISPR/Cas9-mediated genetic knockout (Fig. 1c). Both methods resulted in a significant reduction in cancer-associated fibroblast migration and HMEC-1 tubulogenesis (Fig. 1d). These findings correlate with the functional role of SLC5A3 in cell regulation and tumorigenesis and given its identification in circulating plasma, suggest its potential utility as a diagnostic biomarker.

We report SLC5A3 in sEVs for the first time, as it has not been previously linked to pancreatic cancer, disease stage, or clinical outcomes. Additionally, SLC5A3 was found in sEVs from pancreatic cancer patient plasma but lowly expressed in healthy (non-disease) patient samples, suggesting its potential role as a predictive diagnostic for non-invasive early detection and monitoring of late-stage pancreatic cancer.

Elevated SLC5A3 levels in sEVs from PDAC patients suggest a distinct molecular signature that can be leveraged for diagnostic purposes. Furthermore, functional studies demonstrate that SLC5A3 directly influences tumour microenvironment dynamics, including cancer-associated fibroblast migration and angiogenesis, highlighting its biological relevance. These findings position SLC5A3 as a potential critical marker for non-invasive early detection through liquid biopsy approaches and as a potential therapeutic target, providing new opportunities to tailor treatments based on specific molecular features of PDAC.

In addition, our findings can focus further research into the mechanistic roles of SLC5A3 in pancreatic cancer and as a signalling mediator through EVs. Understanding how SLC5A3 contributes to tumour aggressiveness can uncover new pathways and targets for therapeutic intervention, ultimately leading to better clinical outcomes for patients.

Our work demonstrates that sEVs contain not only the inositol transporter SLC5A3 but also the entire molecular machinery (Supplementary Information) necessary to produce key inositol metabolites, such as phosphatidylinositol 4,5-bisphosphate, a critical substrate for essential second messengers. More importantly, our findings suggest that sEVs are not merely organelles involved in cargo delivery but function as hubs for producing signalling molecules within target cells.

## Supplementary information

Supplementary Material

## Data Availability

Acquired NTA data for all samples is provided in the data repository (MassIVE with identifier MSV000097265). MS-based proteomics data (RAW, parameter, and result in protein identification and differential expression analyses) are deposited to the ProteomeXchange Consortium via the MassIVE partner repository and available via MassIVE with an identifier (MSV000097265). We further provide access to mass spectrometry data (RAW) associated with (i) human non-malignant epithelial and pancreatic cancer cell models (PeptideAtlas, #PASS01331) and (ii) human patient-derived plasma EV samples (MassIVE, #MSV000094140).
