# Supplementary material for: Oncogenic proteome of pancreatic cancer extracellular vesicles: sodium/myo-inositol cotransporter as a potential marker
Source: Signal Transduct Target Ther. 2025 May 7;10:148. doi: 10.1038/s41392-025-02232-9 (PMC12056050; doi:10.1038/s41392-025-02232-9)
Supplement: Supplementary file 1 — Supplementary Material [file 41392_2025_2232_MOESM1_ESM.docx]

Supplementary Materials for

Oncogenic proteome of pancreatic cancer extracellular vesicles: sodium/myo-inositol cotransporter as a potential marker

Arunima Panda, Krish Ragunath, Marina Pajic, David W. Greening^#^ and Marco Falasca^#^

Correspondence to: marco.falasca@unipr.it and David.Greening@baker.edu.au

**This file includes:**

Materials and Methods

Materials and Methods

### Cell culture. Patient-derived cell lines TKCC-02, TKCC-05, TKCC-06, TKCC-07, TKCC-15, TKCC-19, TKCC-26 and TKCC-27 were obtained from the Garvan Institute, Sidney (BioResource-PDCLs, Australian Pancreatic Cancer Genome Initiative). The key characteristics of these cell lines are displayed in Figure 1a. PDCLs were cultured as previously described^1^. Human Microvascular Endothelial Cells, HMEC-1, (Code ATCC CRL-3243), were grown in MCDB131 (Life Technologies cat# 10372019) with the addition of 20% FBS, penicillin/streptomycin, L-glutamine 10 mM, hydrocortisone 1 μg/mL, and epidermal growth factor (10 ng/mL). Human Immortalized Pancreatic cancer-associated fibroblasts cells (CAFs), used for the migration assay, were obtained from Neuromics (Edina, MN, USA; PC00B5) and grown in DMEM High glucose (4.5 g/l glucose, Cat# 11965084, Gibco) supplemented with 10% FBS, 1% PS and 10nM glutamine. MIA PaCa-2 cells (Code ATCC CRL-1420) were cultured in DMEM High glucose (4.5 g/l glucose, Cat# 11965084, Gibco) plus 10% FBS and 2.5% horse serum (Cat# 26050088, Gibco).

### Isolation of cell-derived small extracellular vesicles. sEVs were isolated from PDCLs TKCC-02, TKCC-05, TKCC-06, TKCC-07, TKCC-15, TKCC-19, TKCC-26 and TKCC-27 as described ^2^. After at least two passages in T75 flasks, EV-depleted FBS (Thermo Fisher Scientific, Cat# A2720301) was substituted to the respective medium of culture. Cells were then transferred in T175 flasks (Thermo Fisher Scientific, Cat# 159910) and incubated for 72 hours at 37^o^C. Media was then centrifuged at 450 x g for 5 min, and at 2000 x g for 10 min at 4^o^C to remove cell debris and apoptotic vesicles. The media was then filtered using a 0.22 µm PVDF filter (Cat# SLGV033R Sigma Aldrich) before being spun at 120,000 x g at 4^o^C for 2 hours using the Optima XE ultracentrifuge (Beckman). The resulting pellet was resuspended in PBS and re- ultracentrifuged (120,000 x g at 4^o^C for 2 hours). The final pellet, resuspended in 30 µl of PBS, was stored at -20^o^C for no longer than one week.

Isolation of blood-derived small extracellular vesicles. Ten ml of blood were collected from pancreatic cancer patients (in collaboration with the Royal Perth Hospital) and healthy volunteers in BD Vacutainer® K2 EDTA Tubes (Cat# BDAM367862, Avantor). The inclusion criteria for this study were age between 45-75yrs, no history of cancer or diabetes for the healthy controls, a minimum of 6hrs of fasting before blood collection. Patient P1 was initially suspected to have pancreatic cancer but was later diagnosed with chronic pancreatitis. The blood was immediately centrifuged at 1000 x g for 10 min, the supernatant was transferred into a 15 ml falcon tube and centrifuged at 2500 x g for 15 min twice using different tubes. The obtained supernatant was then distributed into microcentrifuge tubes (Cat# 3453PK, Thermo Fisher Scientific) and centrifuged at 4°C for 20 min before being filtered with a 0.22µm PVDF filter into a new 15ml Falcon tube. The plasma was then stored at -80 until the final EV extraction was performed using the same protocol as described above.

### Western Blotting. Protein quantification for all sEVs and cells samples was performed using the Micro BCA Protein Assay Kit (Thermo Fisher Scientific). 5µg and 30µg of proteins were loaded for sEVS and cells respectively into a 10% polyacrylamide gel and electrophoresis was run at 100V. Membranes were then incubated with primary antibodies, Calnexin, TSG101, GM130, GAPDH, alpha-actin, CD82, and CD63, all diluted 1:1000 in 3% BSA in TBS-T (Cell Signaling Technology), and SLC5A3, dilution 1:5000 (Abcam), at 4^o^C overnight. The next day, membranes were rinsed 3 times for 10 min in TBST at room temperature before being incubated with secondary anti-mouse IgG, HRP-linked antibody (Cell Signaling Technology #7076) or anti-rabbit IgG, HRP-linked antibody (Cell Signaling Technology #7074), 1:40,000 dilution in BSA 3% TBS-T for 1 hour at room temperature. 3 washes of 10 min with TBST at RT and one of 5 min in 1X TBS followed, before membranes’ incubation with Clarity Western ECL Blotting Substrates (Bio-Rad) for 5 mins. Results were visualised using the ChemiDoc MP Imaging System (BioRad).

Scanning Electron Microscopy. An equal volume of 5% glutaraldehyde (Sigma-Aldrich) was added to the sEV samples previously resuspended in PBS. The samples were transferred to a glass slip and incubated overnight at room temperature. The next day, serial dehydrations with 10, 20, 40 and 90% solutions of ethanol were performed before mounting the glass slip on an aluminium stub using carbon conductive tape. Scanning Electron microscopy (SEM) was performed using the Zeiss Neon 40EsB (ZEISS NEON 40EsB FIBSEM), a dual-beam field emission scanning electron microscope (EHT 10 kV Aperture Size 20μm). Imaging was performed at the Microscopy and Microanalysis Facility at the Curtin University John de Laeter Centre, Faculty of Science and Engineering.

### Nanoparticle Tracking Analysis. To determine particle concentration and size, nanoparticle tracking analysis (NTA, ZetaView, Particle Metrix, PMX-120; 405 nm laser diode) was used. Sample volume was normalized, and analysis was performed in triplicate with samples diluted in 1 mL of PBS (14190-144, Thermo Fisher Scientific). The parameters used were camera sensitivity: 80, min area: 5, max area: 1000, brightness: 30, min trace length: 15, temperature: 25 ◦C. 30 frames for 11 positions (medium video setting) were used to capture images. The instrument was set up with calibration beads (Nano FCM, S16M-Exo). Data were analysed using the ZetaView software 8.5.10. Acquired NTA data for all samples is provided in data repository (MassIVE with identifier MSV000097265).

### sEVs uptake assay. The PKH67 Green Fluorescent Cell Linker Kit (Cat# PKH67GL, Sigma Aldrich) for general cell membrane labelling was used to verify sEV incorporation into recipient cells. 45,000 HMEC-1 cells/well were seeded in a 6-well plate and incubated for 24 hours at 36 degrees and treated the day after with TKCC-05 and TKCC-02-derived sEVs according to the kit protocol. The results were visualised with the fluorescent microscope Olympus 1X-51 inverted and cellSens software at 20X.

Mass Spectrometry-Based Proteome Profiling. Proteome profiling was performed as described ^2^. Samples were quantified with microBCA, as mentioned above, in biological quadruplicates. The 10 µg protein samples were lysed in SDS sample buffer. Proteins were then denatured at 95 °C for 5 min. Proteins were separated by SDS-PAGE (150 V, 15 min) and then visualized through Imperial Protein Stain (Thermo Fisher Scientific). Each lane was cut into two gel pieces, reduced with 2 mM tri(2-carboxyethyl)phosphine hydrochloride (Sigma-Aldrich, C4706) at 22 °C for 4 h with gentle shaking, followed by alkylation with 25 mM iodoacetamide in the dark for 30 min at 25 °C. they were then digested with trypsin (Promega, V5111) at a 1:50 enzyme : substrate ratio at 30 °C for 16 h. This peptide mixture was acidified to a final concentration of 2% formic acid and 0.1% trifluoroacetic acid and centrifuged at 16 000 x g for 15 min. Next, peptides were purified and extracted using reverse-phase C18 StageTips (Sep-Park cartridges, Waters, MA) in 85% v/v acetonitrile (ACN) in 0.5% v/v formic acid (FA). Peptides were then freeze-dried and acidified with buffer containing 0.1% FA & 2% ACN. Proteomic experiments were undertaken in biological quadruplicates with their respective cells (n = 2) with MIAPE-compliance. A nanoflow UPLC instrument (Ultimate 3000 RSLCnano, Thermo Fisher Scientific) was linked to a Q-Exactive HF Orbitrap mass spectrometer (Thermo Fisher Scientific) with a nano electrospray ion source (Thermo Fisher Scientific). Peptides were loaded (Acclaim PepMap 100, 5 mm x 300 µm i.d., µ-Precolumn packed with 5 µm C18 beads, Thermo Fisher Scientific) and separated (BioSphere C18 1.9 µm 120 A, 360/75 µm x 400 mm, NanoSeparations) with a 120 min gradient from 2 – 100% v/v phase B (0.1% v/v FA in 80% v/v ACN) (2 – 100% 0.1% FA in ACN (2 – 40% from 0 – 100 min, 40 – 80% from 100 – 110 min, at a flow rate of 250 nL min^-1^ operated at 55 °C. Mass spectrometry was operated data-dependently, with the top 10 most abundant precursor ions in the survey scan (350 – 1500 Th) being chosen for MS/MS fragmentation. Survey scans existed at a resolution of 60 000, with an MS/MS resolution of 15 000. Unassigned precursor ion charge states and singly charged species were not accepted, with peptide match turned off. The isolation window occurred at 1.4 Th with selected precursors fragmented by higher energy collisional dissociation (HCD) with normalized collision energies of 25 with a maximum ion injection time of 110 ms. Ion target values were 3e6 for survey, and 1e5 for MS/MS scans. Dynamic exclusion occurred for 30 s. Data were acquired using X calibur software v4.0 (Thermo Fisher Scientific). For this study, MS-based proteomics data (RAW, parameter, and result protein identification and differential expression analyses) are deposited to the ProteomeXchange Consortium via the MassIVE partner repository and available via MassIVE with identifier (MSV000097265). We further provide access to mass spectrometry data (RAW) associated with (i) human non-malignant epithelial and pancreatic cancer cell models (PeptideAtlas, #PASS01331)^2^ and (ii) human patient derived plasma EV samples (MassIVE, #MSV000094140).

Database searching and protein identification. Raw data was pre-processed as previous groups performed ^3^ and processed through MaxQuant ^4^ (v1.6.0.1) with Andromeda (v1.5.6), using the Human-only (UniProt #71 785 entries) sequence database (January 2018). Genes and proteins were searched for as described ^5^ with a precursor ion tolerance of 10 parts per million at a fragment tolerance of 0.5 Da and minimum peptide length of 6, with a false discovery rate <1% at the peptide and protein levels. Tryptic digestion occurred for up to two missed cleavages, with cysteine carbamidomethylation utilized for fixed modification. Methionine oxidation and protein N-terminal acetylation were harnessed as variable modifications. Finally data was analysed with label-free quantification (LFQ) ^6^. LFQ intensities for all unique and razor peptides are included, with zero intensity values replaced with a constant value of 1 (imputation, constant value) to calculate fold change ratios. As a result, LFQ intensity values are averaged and normalized for protein length, with fold change ratios calculated. Contaminants, and reverse identification were excluded. Protein identifications were accepted, if they reached greater than 99% probability (protein false discovery rate < 1%) and contained at least two identified unique peptides (*q-*values < 1%). *P-*values were adjusted by the Benjamin-Hochberg multi-test adjustment method for a higher number of comparisons as performed^7^, and statistics performed as previously described^8^. For pathway analyses, Kyoto Encyclopedia of Genes and Genomes (KEGG) and NIH Database for Annotation, Visualization and Integrated Discovery Bioinformatics Resources 6.7 (DAVID) resources were utilized using recommended analytical parameters^9^. STRING protein-protein interaction network was used for protein association pathways and interaction mapping, in addition to KEA3 (Kinase Enrichment Analysis) used to understand protein interactors and regulatory signalling targets in a specific protein set (SLC5A3). UniProt database resource (www.uniprot.org) was utilized for gene ontology (GO) enrichment. Network analysis occurred using biological process and molecular function. For the above pathway and enrichment analyses, *Homo Sapiens* background genome was utilized. Human Protein Atlas was employed for cancer tissue expression analysis (RNA/protein) ^10^. Differentially expressed proteins were identified if fold change ratios were greater than or equal to 2.0, with *P-*values of less than 0.05, with identifications in at least two out of four biological sample replicates. Clustering of samples were performed by principal component analysis (PCA) and visualized using ggplot2 and ggfortify^11^ (https://cran.r-project.org/web.packages/ggfortify/index.htlm). The heat map of proteins was performed using gplots (<https://cran.r-project.org/web/packages/gplots/index.html>). In our proteomics analysis of PDCL cell lines, we identified a set of proteins involved in inositol metabolism that were uniquely expressed in sEVs but absent in the corresponding cells. These proteins include PIP4K2A, PIP5K1A, SLC5A3, INPP5A, and PLD1, suggesting a selective enrichment of inositol metabolism-related components in EVs.

### Migration Assay. CAF cells were seeded onto a 96-well plate and incubated for 24h or until 100% confluency was reached. The medium was then replaced with medium containing EV-depleted FBS (Thermo Fisher Scientific, Cat# A2720301). After cleaning the scratch wound device by soaking it for 5 min in 45 ml of sterile water and 45 ml of 70% ethanol, the scratch was made (and repeated if necessary). After rinsing with PBS, the wells were treated in triplicates with PBS as a control, and 0.125µg/ml of sEVs from TKCC-05. Cell migration was assessed upon stimulation with MIA PaCa-2 sEVs (0.05ug/ul) or TKCC-05 at the same concentration, with the SLC5 family inhibitor phlorizin (10µM), sEVs plus phlorizin, and PBS as a control. Plates were incubated in the Operetta CLS live-cell analysis system incubator, with images obtained every hour for a period of 24 hours and analysed with the Operetta CLS live-cell analysis system automated software.

### Angiogenesis assay. HMEC-1 cells (ATCC CRL-3243) were used for this experiment and treated with PBS only, MIA PaCa-2-derived sEVs (0.05 µg/μl), SLC5 family inhibitor phlorizin 10 μM concentration, and a combination of sEVs and phlorizin. Wells of a 96-wells plate were coated with 25-30 µl/well of thawed Matrigel matrix solution (Growth Factor Reduced Basement Membrane Matrix, Corning, #FAL356231) and maintained on ice throughout the process using cold pipette tips. HMEC-1 cells were counted and incubated at 37^o^C for 1 hour in serum-free HMEC-1 medium in a falcon tube. The Matrigel was evenly spread in each well with the head of a 1ml syringe plunger and incubated at 37^o^C for 30 min. After incubation, 15,000/well HMEC-1 cells were seeded on top of the Matrigel solution in 200ul/well of serum-free medium and incubated for 1 hour before removing the old media and applying the treatments; then, they were placed into the Incucyte® Live-Cell Analysis System, for 24 hours. Images were analysed with a double-blind quantitative analysis by manually counting the number of branching points in the tubule formation.

CRISPR-Cas9 SLC5A3 KO. We generated SLC5A3 wild-type (WT) and knockout (KO) clones using CRISPR/Cas9 gene editing. Clonal selection was performed, followed by validation of gene knockout efficiency through qPCR and Western blot analysis. To confirm the absence of off-target effects and verify differential gene expression between SLC5A3 WT and KO clones, we performed RNA sequencing. SMIT CRISPR/Cas9 KO Plasmid (h) and SMIT HDR Plasmid (h) (Cat# sc-403864 and sc-418922, Santa Cruz Biotechnology) were used for transfection. Control CRISPR/Cas9 Plasmid with HDR Plasmid was used for the control cell line. Transfected TKCC05 cells underwent Puromycin selection (0.5 µg/ml) (Cat# P8833 Sigma-Aldrich).

Statistical analysis. Data are presented as mean ± SEM, and statistical analysis was conducted across a minimum of 3 independent experiments using the GraphPad PRISM V10.0 software. Statistical tests employed include one-way ANOVA for migration and angiogenesis assays. A 95% confidence interval was used, and statistical significance was set at P < 0.05. Proteomic experiments were undertaken in biological quadruplicates with their respective cells (n = 2) with MIAPE-compliance^12^.

**References**

1 Chou, A. *et al.* Tailored first-line and second-line CDK4-targeting treatment combinations in mouse models of pancreatic cancer. *Gut*. **67**, 2142-2155, (2018).

2 Emmanouilidi, A., Paladin, D., Greening, D. W. & Falasca, M. Oncogenic and Non-Malignant Pancreatic Exosome Cargo Reveal Distinct Expression of Oncogenic and Prognostic Factors Involved in Tumor Invasion and Metastasis. *Proteomics*. **19**, e1800158, (2019).

3 Gorshkov V, V.-B. T., Kjeldsen F. SuperQuant: A Data Processing Approach to Increase Quantitative Proteome Coverage. *Anal Chem*. **87**, 6319-6327, (2015).

4 Cox J, M. M. MaxQuant enables high peptide identification rates, individualized p.p.b.-range mass accuracies and proteome-wide protein quantification. *Nat Biotechnol*. **26**, 1367-1372, (2008).

5 Gopal SK, G. D., Mathias RA, Ji H, Rai A, Chen M, et al. YBX1/YB-1 induces partial EMT and tumourigenicity through secretion of angiogenic factors into the extracellular microenvironment. *Oncotarget*. **6**, 13718-13730, (2015).

6 Luber CA, C. J., Lauterbach H, Fancke B, Selbach M, Tschopp J, et al. Quantitative proteomics reveals subset-specific viral recognition in dendritic cells. *Immunity*. **32**, 279-289, (2010).

7 Yoav B, Y. H. Controlling The False Discovery Rate - A Practical And Powerful Approach To Multiple Testing. *Journal of the Royal Statistical Society*. **57**, 289-300, (1995).

8 Tauro BJ, M. R., Greening DW, Gopal SK, Ji H, Kapp EA, et al. Oncogenic H-ras reprograms Madin-Darby canine kidney (MDCK) cell-derived exosomal proteins following epithelial-mesenchymal transition. *Mol Cell Proteomics*. **12**, 2148-2159, (2013).

9 Huang DW, S. B., Lempicki RA. Systematic and integrative analysis of large gene lists using DAVID bioinformatics resources. *Nat Protoc*. **4**, 44-57, (2009).

10 Uhlen, M. *et al.* A pathology atlas of the human cancer transcriptome. *Science*. **357**, (2017).

11 L., W. ggplot2: Elegant Graphics for Data Analysis by WICKHAM, H. *Biometrics*. **67**, 2011, (2011).

12 Taylor CF, P. N., Lilley KS, Binz P, Julian RK, Jones AR, et al. he minimum information about a proteomics experiment (MIAPE). *Nat Biotechnol*. **25**, 887-893, (2007).
